# Intrarenal Doppler ultrasonography in patients with HFrEF and acute decompensated heart failure undergoing recompensation

**DOI:** 10.1007/s00392-023-02184-6

**Published:** 2023-03-25

**Authors:** M. Wallbach, M. Valentova, M. R. Schroeter, A. Alkabariti, I. Iraki, A. Leha, D. Tampe, G. Hasenfuß, M. Zeisberg, K. Hellenkamp, M. J. Koziolek

**Affiliations:** 1 Department of Nephrology and Rheumatology, University Medical Center Göttingen, Georg-August-University Göttingen, Robert-Koch-Str. 40, 37075 Göttingen, Germany; 2 Department of Cardiology and Pneumology, University Medical Center Göttingen, Georg-August-University Göttingen, Göttingen, Germany; 3 German Center for Cardiovascular Research (DZHK), Partner Site, Göttingen, Germany; 4 Deutsches Herzzentrum Göttingen, Göttingen, Germany; 5 Deutschen Gesellschaft Für Kardiologie (Young DGK), University Medical Center, Göttingen, Germany; 6 Department of Medical Statistics, University Medical Center, Göttingen, Germany

**Keywords:** Cardiorenal syndrome, Acute decompensated heart failure, Renal venous congestion, Renal function, Albuminuria

## Abstract

**Objectives:**

Renal venous congestion due to backward heart failure leads to disturbance of renal function in acute decompensated heart failure (ADHF). Whether decongestion strategies have an impact on renal venous congestion is unknown. Objective was to evaluate changes in intrarenal hemodynamics using intrarenal Doppler ultrasonography (IRD) in patients with heart failure with reduced ejection fraction (HFrEF) and ADHF undergoing recompensation.

**Methods:**

Prospective observational study in patients with left ventricular ejection fraction (LV-EF) ≤ 35% hospitalized due to ADHF. IRD measurement was performed within the first 48 h of hospitalisation and before discharge. Decongestion strategies were based on clinical judgement according to heart failure guidelines. IRD was used to assess intrarenal venous flow (IRVF) pattern, venous impedance index (VII) and resistance index (RI). Laboratory analyses included plasma creatinine, eGFR and albuminuria.

**Results:**

A number of 35 patients with ADHF and LV-EF ≤ 35% were included into the study. IRD could be performed in 30 patients at inclusion and discharge. At discharge, there was a significant reduction of VII from a median of 1.0 (0.86–1.0) to 0.59 (0.26–1.0) (p < 0.01) as well as improvement of IRVF pattern categories (p < 0.05) compared to inclusion. Albuminuria was significantly reduced from a median of 78 mg/g creatinine (39–238) to 29 mg/g creatinine (16–127) (p = 0.02) and proportion of patients with normoalbuminuria increased (p = 0.01). Plasma creatinine and RI remained unchanged (p = 0.73; p = 0.43).

**Discussion:**

This is the first study showing an effect of standard ADHF therapy on parameters of renal venous congestion in patients with HFrEF and ADHF. Doppler sonographic evaluation of renal venous congestion might provide additional information to guide decongestion strategies in patients with ADHF.

**Graphical abstract:**

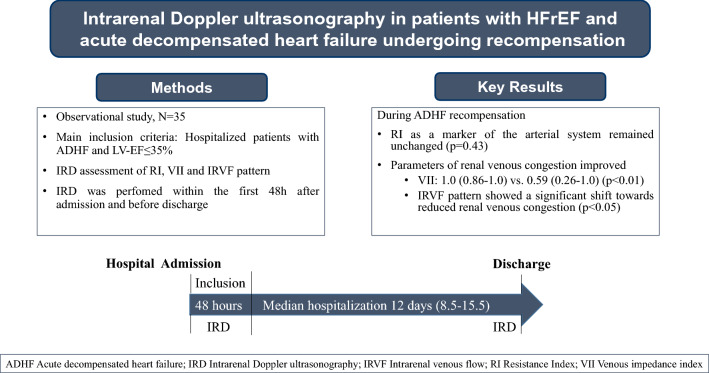

## Introduction

The cardiorenal syndrome describes the interaction between heart and kidney and was classified into five subtypes in 2008 [1]. In acute decompensated heart failure (ADHF), classified as cardiorenal syndrome type 1, deterioration of renal function is very common and is associated with adverse clinical outcomes [1–3]. Elevation of right atrial and central venous pressure (CVP) is transmitted to the renal veins leading to an increased interstitial and tubular hydrostatic pressure within the encapsulated kidney, which consequently decreases the glomerular filtration rate (GFR) [4]. Whereas renal function is relatively robust against reduced cardiac output, increased CVP leading to renal congestion has been one of the main pathophysiologic findings in cardiorenal syndrome [5]. Hence, it is obvious that an adequate control of the congestion state combined with preservation of renal function is a central goal in the management of heart failure (HF). Beyond CVP as a surrogate for congestion, there is upcoming evidence that parameters of renal hemodynamics are superior in determination of renal venous congestion and renal function. [6–8] However, the well-established resistance index (RI) which correlates with renal function, pathophysiology, and prognosis in both renal and cardiac disease was inferior in the prediction of outcome and diuretic response in HF compared to intrarenal venous flow (IRVF) pattern or venous impedance index (VII), which represent parameters of renal venous flow [6, 7, 9]. Thereby, in patients with heart failure with reduced ejection fraction (HFrEF) volume expansion leads to a significant change in renal venous flow, even before changes in cardiac filling pressure becomes evident [6]. In ADHF congestion contributes to renal dysfunction, neurohormonal activation, increased intra-abdominal pressure, excessive tubular sodium reabsorption, fluid overload and diuretic resistance [10]. In some refractory cases, it is necessary to treat patients with renal replacement therapy (RRT) to overcome diuretic resistance and to treat acute kidney injury [11]. Effective decongestion strategies might preserve renal function and relief symptoms of acute decompensation. Though there is growing evidence that Doppler sonographic parameters might be associated with the prognosis in HF, response to volume expansion as well as diuretic therapy [6, 7, 12], no data are available for hospitalized patients with HFrEF undergoing standard decongestion therapy. Therefore, the objective of the present study was to analyze VII and IRVF pattern as Doppler sonographic parameters of renal venous congestion in patients with HFrEF and ADHF and to determine whether recompensation has a meaningful impact on these renal venous congestion parameters.

## Methods

### Study design

This was a prospective, single center, observational study in a university hospital between April 2020 and April 2022. Subjects were eligible for study inclusion if (1) they were ≥ 18 years of age, (2) were able to give informed consent, (3) had a clinical diagnosis of ADHF (signs of congestion, e.g. orthopnea, rales, peripheral edema, jugular venous distension, pulmonary edema or pleural effusion) with echocardiographic signs of an impaired left ventricular ejection fraction (LV-EF) ≤ 35% at the time of inclusion and (4) were hospitalized for HF. Exclusion criteria were as follows: (1) patients with left ventricular device or after heart transplantation, (2) prior renal replacement therapy, prior kidney transplantation or need for RRT at screening, (3) known precapillary pulmonary hypertension, (4) known high-grade stenosis of the aortic valve, (5) chronic liver dysfunction with ascites, (6) circumstances which hinder performance or interpretation of IRD or (7) pregnancy. IRD measurements and laboratory analysis were performed within the first 48 h after admission and in a stable condition before discharge. Medical history, outpatient medications, clinical findings, and laboratory values were extracted from clinical records. Treatment of ADHF was based on current HF guidelines [13, 14]. Laboratory analyses included plasma creatinine, determination of eGFR (CKD-EPI creatinine equation) and albuminuria. The study complies with the principles of the Declaration of Helsinki and local ethical committee approved the study protocol (Ethical vote number 38/2/19). All patients provided written informed consent.

### Intrarenal Doppler ultrasonography (IRD)

Patients underwent IRD assessment as previously described. [6, 7, 15]. IRD was performed as a part of clinical routine in our department with the use of a standard convex transducer for abdominal sonography with a frequency range 1–6 MHz. IRD parameters of the right kidney were used for analysis with each subject in the left semi-lateral decubitus position. When there was no sufficient image quality on the right side, parameters of the left kidney were analyzed. Color Doppler images were used to determine interlobar vessels. The renal resistance index (RI) at an interlobar artery was calculated as follows RI = (Vmax − Vmin)/Vmax [6]. The venous impedance index (VII) was calculated for the venous flow in analogues to the RI in the arterial system as VII = (Vmax − Vmin)/Vmax [6]. In addition, Doppler waveforms of the venous flow were divided into 4 flow patterns as described before [8]: Continuous venous flow (no congestion, stage 0), pulsatile flow (stage 1 congestion), biphasic flow (stage 2 congestion), and monophasic flow (stage 3 congestion). Congestion stage 1 to 3 were defined as discontinuous flow which was considered abnormal showing a venous flow at nadir of zero or even a positive venous flow value. Consequently, if the nadir of the venous flow was zero or positive, the VII was defined as 1. Therefore, VII ranged between 0 and 1. All values were recorded as means of at least three measurements in different interlobar vessels within the kidney. If atrial fibrillation was present, an index beat (the beat following 2 preceding cardiac cycles of equal duration) was used for each measurement [8].

### Laboratory data and renal function

Plasma creatinine, NT-pro BNP and albuminuria were routinely analyzed by standard methods in central laboratory of the University Medical Center Goettingen at inclusion and before discharge from hospital. eGFR was calculated using the CKD-EPI creatinine equation. Urine samples were collected, centrifuged at 1000×*g* for 10 min at 4 °C to remove cell debris and casts. Worsening renal function (WRF) was defined as an increase in plasma creatinine of ≥ 0.3 mg/dl during hospitalization [16].

### Statistical analysis

The data were analyzed using the statistical software SPSS Version 28.0.1.0 (IBM Corp., Armonk, NY, USA) and Graphpad Prism Version 9 (GraphPad Software, San Diego, CA, USA). To assess time-dependent changes in the investigated variables, paired two-sided t-test or Wilcoxon signed rank test were used, where appropriate. Differences between subgroups were compared by an independent t-test or Mann–Whitney U test for continuous variables or the Pearson’s chi square test or fisher’s exact test for categorical values. Results are expressed as mean ± SD, median (IQR) or as a number with percentage for categorical variables. With respect to albuminuria and NT-pro BNP levels, significance was tested after logarithmic transformation (base-10), as a log-normal distribution was assumed. The threshold for statistical significance was chosen to be p < 0.05. Two observers (MW and MK) independently assessed RI, VII and IRVF patterns in 15 patients sequentially. To test intrarater reliability, a single observer (MW) analyzed data of 15 patients in a blinded manner twice separated by a 1-month interval. Agreement between the different raters as well as intrarater agreement was calculated with intraclass correlation coefficients (ICC) [mean and 95% confidence interval (CI)] using two-way mixed effects as model and absolute agreement as type [17].

## Results

### Study population

A total of 35 patients with ADHF and reduced LV-EF ≤ 35% were included. Patients' mean age was 68 ± 16 years and 31% were female. Mean body mass index (BMI) was 30.2 ± 7.4 kg/m^2^. According to baseline values the present cohort included 11 patients (31%) with eGFR < 30 ml/min/1.73 m^2^. Diabetes mellitus was present in 15 patients (43%). In average patients took 3.3 ± 1.6 classes of HF medications at admission. Baseline characteristics are listed in Table 1.

**Table 1:** Patients’ characteristics at inclusion

N	35
Female n (%)	11 (31%)
Age (years)	68 ± 16
BMI (kg/m^2^)	30.2 ± 7.4
eGFR (ml/min/1.73m^2^)	53.9 ± 26.2
Albuminuria (mg/g creatinine)	78 (39–238)
Relevant concomitant diseases	
CAD	25 (71%)
Atrial fibrillation	22 (63%)
Arterial hypertension	24 (69%)
Hyperlipoproteinemia	19 (54%)
Obesity (BMI ≥ 30 kg/m^2^)	17 (49%)
Diabetes mellitus	15 (43%)
History of smoking	14 (40%)
Hydration status	
Peripheral edema	27 (77%)
Pleural effusion (clinical/radiographic signs)	21 (60%)
Dyspnea NYHA IV	34 (97%)
Cardiovascular medication	
ACE-inhibitor	7 (20%)
ARB	4 (11%)
ARNI	18 (51%)
Aldosteron receptor antagonist	21 (60%)
Beta-blocker	26 (74%)
Calcium-channel blocker	3 (9%)
Loop diuretic	26 (80%)
Thiazide	4 (11%)
SGLT2-I	8 (23%)

Values are mean ± SD, n (%), or median (IQR), chronic kidney disease (CKD). Coronary artery disease (CAD), heart failure with reduced ejection fraction (HFrEF), angiotensin receptor blocker (ARB), angiotensin receptor neprilysin inhibitor (ARNI), sodium-glucose-transporter-2-inhibitor (SGLT2-I), left ventricular ejection fraction (LV-EF)

### Volume status, hospitalization and outcome

At inclusion, 27 patients (77%) showed peripheral edema, 21 patients (60%) had clinical or radiographic signs of pleural effusion, 34 patients (97%) had dyspnea at rest. Whereas the majority of patients had known HF diagnosis, in 4 patients (11%) HF was newly diagnosed. Patients were hospitalized for a median of 12 days (8.5–15.5). Decongestion therapy resulted in a significant body weight reduction of − 6.8 ± 5.5 kg (p < 0.01) as well as a median reduction in NT-pro BNP level of − 3266.3 ng/l (− 8932.8 to (− 168.4)) (p < 0.01). Six patients (17%) experienced clinically meaningful adverse events during hospitalization (dead or need for RRT). In five patients (14%; one of them died) RRT was necessary during hospital stay. Two patients (6%) died during hospital stay due to cardiogenic shock.

### Doppler sonographic parameters of renal venous congestion

**Fig. 1:**
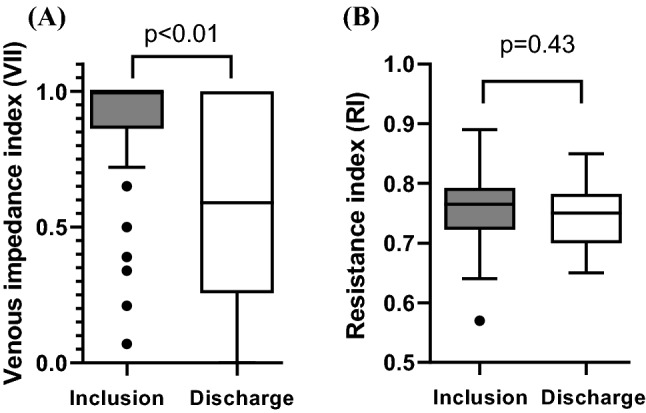
**A** Venous impedance index (VII) and **B** resistance index (RI) at inclusion and discharge. Lines represent median, boxes represents lower and upper quartile, whiskers represent 1.5 × IQR and dots indicate outliers

**Fig. 2:**
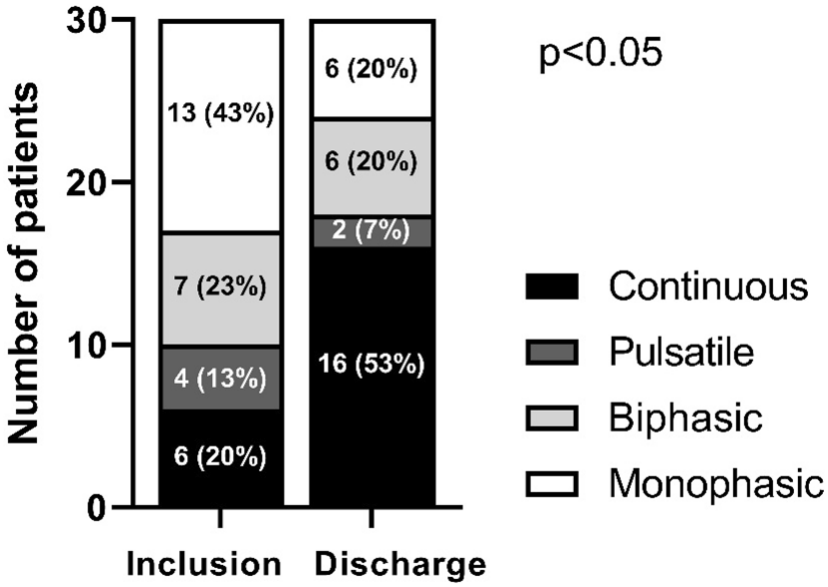
Distribution of IRVF pattern at inclusion and discharge. Values indicate absolute numbers of patients (%)

A total of 5 patients (14%) were excluded from Doppler analysis due to missing discharge data (2 patients (6%) died, 3 patients (9%) have been discharged prior to follow-up Doppler sonography), so that IRD was performed at inclusion and discharge in 30 patients. Regarding the renal arterial system, RI in the interlobar artery did not changed significantly from inclusion to discharge (0.76 ± 0.07 vs. 0.75 ± 0.05, p = 0.43). Compared to admission, VII was significantly reduced at discharge (1.0 (0.86–1.0) vs. 0.59 (0.26–1.0), p < 0.01) (Fig. 1). Stratification according to IRVF pattern showed a significant shift towards reduced renal venous congestion pattern at discharge (p < 0.05) (Fig. 2). At discharge, 15 patients (50%) exhibit an improvement of IRVF pattern and 16 patients (43%) showed continuous venous flow, which was considered as normal IRVF pattern. At admission 6 ADHF patients (20%) showed continuous flow pattern with a median VII of 0.365 (0.243–0.473), accompanied by a median NT-pro-BNP level of 4262 ng/l (1514–5837). There was no difference in patients with improved IRVF pattern in respect to the change in body weight during hospitalization (− 8.3 ± 5.4 kg) compared to patients with no improvement of IRVF (-6.2 ± 5.7 kg) (p = 0.33).

### Association of IRVF pattern with clinical and laboratory parameters

**Fig. 3:**
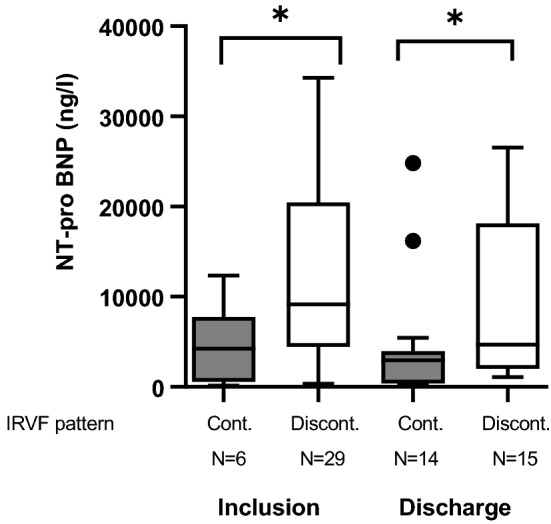
NTpro-BNP levels at inclusion and discharge (B) according to intrarenal venous flow (IRVF) pattern at each time point. Continuous IRVF (no congestion, stage 0), discontinuous IRVF (stage 1–3 congestion). Lines represent median, boxes represents lower and upper quartile, whiskers represent 1.5 × IQR and dots indicate outliers

Patients with continuous IRVF pattern (no congestion) had lower median levels of NT-pro BNP compared to patients with discontinuous IRVF pattern (stage 1–3 congestion) at inclusion (4261.6 ng/l (747.8–6214.8) N = 29 vs. 9152.7 ng/l (4499.2–19,590.9) N = 6) (p < 0.05) and at discharge (2966 ng/l (381.2–3952.0) N = 14 vs. 4682.0 ng/l (2025.0–18,109.0) N = 15 (p = 0.03) (Fig. 3)*.* Patients with continuous IRVF pattern at inclusion also showed numerically lower levels of plasma creatinine compared to patients with discontinuous IRVF pattern (1.21 ± 0.41 mg/dl vs. 2.08 ± 1.02 mg/dl; p = 0.05; patients with need of RRT were excluded). There was no significant difference in the occurrence rate of WRF between patients with continuous (4/6 (67%)) and discontinuous IRVF pattern (13/29 (45%)) at admission (p = 0.40). Accordingly, there was no significant difference in the VII at admission between patients with WRF during hospitalization and patients without WRF (p = 0.86). Patients with monophasic IRVF pattern (stage 3 congestion) at inclusion showed numerically greater weight loss during recompensation (− 8.5 ± 5.3% vs. − 5.3 ± 4.5%, p = 0.10). A higher rate of clinical events (dead or need for RRT) was observed in patients with monophasic and biphasic IRVF pattern (stage 2 and 3 congestion) (N = 6/20, 30%) at inclusion compared to patients with pulsatile (stage 1 congestion) and continuous IRVF pattern (N = 0/15, 0%) (p = 0.03). All 6 patients who died or required RRT exhibited biphasic or monophasic IRVF pattern (2 biphasic, 4 monophasic) consistently accompanied with a VII of 1.

### Renal function and albuminuria

At inclusion, all patients had signs of renal functional impairment with a eGFR below 90 ml/min/1.73 m^2^ and/or occurrence of albuminuria (analysis of albuminuria was available at inclusion in 31 patients). In particular, 33 patients (94%) showed eGFR below 90 ml/min/1.73 m^2^ and 24 of 31 patients (77%) showed albuminuria > 30 mg/g creatinine at inclusion. Two patients (6%) had eGFR ≥ 90 ml/min/1.73 m^2^, 10 patients (29%) had eGFR 30–59 ml/min/1.73 m^2^, 12 patients (34%) had eGFR 30–59 ml/min/1.73 m^2^, 8 patients (23%) eGFR 15–29 ml/min/1.73 m^2^ and 3 patients (9%) had eGFR below 15 ml/min/1.73 m^2^. There was no significant change in mean plasma creatinine between inclusion and discharge (1.62 ± 0.93 mg/dl vs.1.66 ± 0.72 mg/dl, p = 0.73). In 17 patients (49%) WRF occurred during hospital stay. A complete data set for albuminuria at inclusion and discharge was available for 26 patients (two patients died (6%), four patients underwent renal replacement therapy (11%), in three patients (9%) no urine collection could be performed). Decongestion therapy resulted in significant reduction in albuminuria from a median of 78 mg/g creatinine (39–238) to 29 mg/g creatinine (16–127) (p = 0.02). In accordance, categories of albuminuria were significantly shifted toward improvement (p = 0.01) (Table 2). Consequently, proportion of patients with at least albuminuria of > 30 mg/g creatinine significantly decreased from 21 patients (81%) at inclusion to 12 patients (46%) at discharge (p = 0.02).

**Table 2:** Changes of renal function, weight and NT-proBNP

	Inclusion	Discharge	P
Parameter of renal function			
Plasma creatinine (mg/dl)^a^	1.62 ± 0.93	1.66 ± 0.72	0.73
eGFR (CKD-EPI creatinine equation)^a^ (ml/min/1.73 m^2a^)	53.9 ± 26.2	47.7 ± 21.1	0.09
Albuminuria (mg/g creatinine)^b^	78 (39–238)	29 (16–127)	0.02
Categories of albuminuria^b^			
< 30 mg/g creatinine	5 (19%)	14 (54%)	0.01
30–300 mg/g creatinine	17 (65%)	7 (27%)	
> 300 mg/g creatinine	4 (15%)	5 (19%)	
WRF during hospitalization	17 (49%)		
Parameter associated with congestion			
Body weight (kg)^d^	91.8 ± 27.6	85.0 ± 26.1	< 0.01
NT-proBNP (ng/l)^c^	8473 (4158–17,524)	3430 (1160–10,741)	< 0.01

Values are mean ± SD, median (IQR) or n (%)

^a^N = 29, patients who need RRT or died where excluded from analysis, ^b^patients where only analyzed if data on albuminuria where available for the inclusion and discharge visit, N = 26, Worsening renal function (WRF) was defined as increase in creatinine of ≥ 0.3 mg/dl during hospitalization. ^c^N = 29, ^d^N = 32

### Intra- and interrater agreement

Intrarater and interrater agreement of RI and VII measurements using ICC were as follows: RI 0.98 (95% CI 0.92–0.99) and 0.94 (95% CI 0.83–0.98), respectively, and VII 0.98 (95% CI 0.94–0.99) and 0.90 (95% CI 0.74–0.97) indicating an overall excellent intra- and interrater reliability [17]. Classification of IRVF pattern were consistent between intra- and interrater assessments.

## Discussion

The present study has three major findings: (1) Patients with ADHF show distinct Doppler sonographic signs of renal venous congestion at admission with high VII and altered IRVF pattern as well as signs of renal functional and/or structural impairment. (2) Decongestive strategies based on standard ADHF therapy contribute to an improvement of VII and IRVF pattern as well as albuminuria, whereas renal RI and plasma creatinine remained unchanged. (3) Patients with higher degree of renal venous congestion at the time of decompensation determined by IRD are more likely to experience clinically meaningful complications such as need for RRT or dead during hospitalization.

There are a few previous trials investigating renal venous congestion in patients with HF using IRD. In the first study by Iida et al., 217 patients with HF were analysed for renal venous congestion [7]. It is remarkable that despite the investigated collective consisted of stable HF patients (N = 71) and patients before discharge after recompensation (N = 151), a large proportion of patients with distinct signs of renal venous congestion was identified (100 patients (46%) with abnormal IRVF pattern—biphasic and monophasic) [7]. Herein, the study could impressively demonstrate that IRVF pattern is able to predict clinical outcome [7]. The second notable study by Nijst et al. investigated renal venous congestion parameters in stable HF patients without signs of volume overload and differentiated between HFrEF and HFpEF [6]. Thereby, volume expansion and consecutive diuretic treatment was performed resulting in an initial increase of VII during volume expansion followed by a decrease of VII after administration of intravenous diuretic therapy [6]. The observed acute decrease of VII 1 h after diuretic administration is in line with the present data showing an effect of standard ADHF therapy on renal venous congestion parameters during hospitalization. A recent study confirmed the prognostic value of discharge IRVF pattern in patients with ADHF [12]. Moreover, a small study showed an association between sonographic parameter of renal venous congestion with creatinine level rise in ADHF [18]. Though there are several similarities with the aforementioned studies such as determined high levels of renal venous congestion parameters in patients with HF, the key differences of the present study is the longitudinal observation analysing the effect of ADHF treatment on IRVF. The present study recruited patients within the first 48 h after admission and thus much earlier in the time continuum of decompensation as compared to previous studies. Subsequently, the effect of decongestion strategies on renal venous congestion in ADHF patients was observed in the present study, showing a clear impact. As previously reported, an eGFR decline during aggressive decongestion in ADHF might paradoxically be associated with a beneficial outcome [19, 20]. Aggressive recompensation strategies do not lead to tubular injury in ADHF, so that moderate impairment of renal function through decongestion strategies must be distinguished from traditional causes of acute kidney injury [21]. Thus, the lack of an improvement of plasma creatinine or eGFR despite an improvement in Doppler sonographic parameters of renal venous congestion in the present study should not automatically lead to a negative interpretation. Moreover, a high proportion of patients in the present study showed WRF during hospitalisation (49%) which is described previously in a comparable way in a prior ADHF trial [21]. In fact, there was a significant reduction in albuminuria as well as an increase in the proportion of patients with normoalbuminuria, indicating a beneficial effect of decongestion therapy on the kidneys.

In the present study, 66% of patients had moderate to severe impairment of renal function and 91% showed abnormal eGFR (< 90 ml/min/1.73 m^2^) at inclusion which is comparable to previous data of the ADHERE database which showed that deterioration of renal function is very common in patients with ADHF [2]. However, there was no significant change in eGFR between admission and discharge in the present study. This might be consequences of the mutually cancelling effects of improving renal venous congestion on the one side and decongestive therapy associated hemodynamic changes leading to a reduction of effective blood volume on the other side.

In line with a previous study that analyzed patients after recompensation of ADHF in which prevalence of WRF was similar in patients with different IRVF patterns at discharge, the present study revealed no significant differences in VII or distribution of IRVF pattern between patients with or without WRF [7]. However, there is even evidence that aggressive decongestion is associated with improved clinical outcomes despite WRF during therapy for ADHF [19, 22]. In accordance to a study that showed volume depending changes in NT-pro BNP levels in stable patients with HFrEF, the present study demonstrated significant reduction of NT-pro BNP levels from inclusion to discharge with higher levels in patients with discontinuous IRVF pattern [6].When interpreting the IRVF pattern at discharge, with 14 patients (47%) showing discontinuous IRVF pattern, which may either indicate incomplete recompensation or insufficient capability of IRD to detect improvement of renal venous congestion, it should be taken into account that patients with HF, even in an apparently stable state, frequently exhibit Doppler sonographic signs of renal venous congestion (30–40%) [6]. Thereby, it must be considered, up to approximately 40% of patients who have been admitted for ADHF have clinical signs of residual congestion at the time of discharge and that this is associated with a higher risk for mortality and rehospitalization [23]. If IRD is sensitive enough to reliably detect improvement of renal venous congestion in ADHF and is suitable for guiding individual treatment of renal congestion, however, remains to be explored. Of note, renal congestion is not present in all patients with ADHF as shown in our study where 6 patients (20%) had continuous IRVF pattern at inclusion. It remains speculative whether those patients exhibited an isolated left-sided cardiac decompensation or whether the sensitivity of the IRD is restricted in ADHF.

In contrast to the observed changes of parameters of the renal venous system, RI as a parameter of the renal arterial system remained unchanged during recompensation, which is consistent with previous data highlighting the predominance of the venous system in cardiorenal syndrome. [5] The present study has several limitations. Due to the observational character of the study, there was a lack of blinding and randomization. Moreover, there was no control group and the sample size was relatively small. The VII can differentiate renal venous congestion in defined spectrum from 0 to 1, but fails to differentiate congestion state in patients with severe renal venous congestion as predominantly seen in the present study at inclusion, where a great proportion of patients exhibited a VII of 1. In this condition, determination of IRVF pattern is apparently superior to differentiate the severity of renal venous congestion. Interpretation of the provided reliability parameters needs some caution, as the investigated ultrasound parameters are considered as markers of congestion for which stability during recompensation and over several weeks cannot be assumed. It must be kept in mind, that renal ultrasound remains a dynamic study with substantial observer-dependency. By interpreting eGFR values it must be kept in mind, that in acute kidney injury, which often occur in ADHF, GFR estimations are not valid. During acute kidney injury and especially with accompanying ADHF, patients are usually not in a stable state and there may be a marked variation in blood pressure and volume status. Moreover, there is a delay between the acute decrease in GFR and increase of serum creatinine and consecutive decrease in eGFR in the acute phase of acute kidney injury. [24, 25] However, baseline creatinine and eGFR values are of clinical interest to characterize the study cohort and to get an approximate impression of the development of renal function during recompensation. A substantial number of HF patients with CKD exhibit renal artery stenosis. [26] In the present study, no patients with known renal artery stenosis were included, however, there was no formal exclusion. In accordance with relevant previous reports investigating IRD in heart diseases [6, 7], in the present study IRD was predominantly performed in the right renal vessels. However, a mean RI of 0.76 ± 0.07 with a lower range of 0.57 in the present cohort, makes the presence of severe, hemodynamic relevant renal artery stenosis rather unlikely. Because of the aforementioned limitations, the present results should therefore be interpreted with caution.

## Conclusion

Recompensation in patients with HFrEF and ADHF resulted in an improvement of the Doppler parameters of renal venous congestion VII and IRVF pattern. Therefore, VII and IRVF pattern may be useful to guide anticongestive strategies in patients with decompensated HFrEF. The evaluation of IRD-guided therapy in larger longitudinal studies in patients with ADHF is needed to evaluate its impact on cardiorenal outcome.

## Data Availability

The dataset analysed for the current study is available from the corresponding author on reasonable request. Applicants will be required to obtain all necessary permissions relevant to data protection regulations before access to data is granted. The data are not publicly available as patients did not agree for their data to be shared publicly.

## Abbreviations

ADHF: Acute decompensated heart failure
CKD: Chronic kidney disease
CVP: Central venous pressure
eGFR: Estimated glomerular filtration rate
HF: Heart failure
HFrEF: Heart failure with reduced ejection fraction
HFpEF: Heart failure with preserved ejection fraction
ICC: Intraclass-correlation-coefficient
IRD: Intrarenal Doppler ultrasonography
IRVF: Intrarenal venous flow
LV-EF: Left ventricular ejection fraction
NT-pro BNP: N-terminal pro–B-type natriuretic peptide
RAAS-I: Renin–Angiotensin–aldosterone-inhibitor
RI: Resistance Index
RRT: Renal replacement therapy
VII: Venous impedance index
